# Association between the triglyceride/high-density lipoprotein (TG/HDL) ratio and incidence of gout: A nationwide cohort study

**DOI:** 10.3389/fendo.2024.1453458

**Published:** 2025-01-10

**Authors:** Yoonkyung Chang, Ju-young Park, Tae-Jin Song

**Affiliations:** ^1^ Department of Neurology, Mokdong Hospital, Ewha Womans University College of Medicine, Seoul, Republic of Korea; ^2^ Department of Applied Statistics, Yonsei University, Seoul, Republic of Korea; ^3^ Department of Statistics and Data Science, Yonsei University, Seoul, Republic of Korea; ^4^ Department of Neurology, Seoul Hospital, Ewha Womans University College of Medicine, Seoul, Republic of Korea

**Keywords:** insulin resistance, triglyceride/high-density lipoprotein ratio, gout, diabetes mellitus, epidemiology

## Abstract

**Introduction:**

The global burden of gout, a severe and painful arthralgia, is of note and is expected to increase in the future. We aimed to investigate the association between the triglyceride/high-density lipoprotein (TG/HDL) ratio, a simple and validated biomarker for insulin resistance, and the incidence of gout in a longitudinal setting in the general population.

**Methods:**

Our study was conducted using the National Health Insurance Service-Health Screening Cohort database of Republic of Korea (2002–2019). We included 300,107 participants who had no previous history of gout and had data for more than three repeated measurements of the triglyceride-glucose (TyG) index. The incidence of gout was determined using at least two or more claims of the ICD-10 code M10.

**Results:**

During a median 9.62 years (interquartile range 8.72–10.53), 14,116 individuals (4.72%) had a reported incidence of gout. In a fully adjusted multivariable time-dependent Cox proportional hazards model with repeated measures of the TyG index, a unit increase in the index significantly increased the risk of gout in the entire cohort (hazard ratio (HR) = 1.150, 95% confidence interval (CI) 1.116–1.184). In a multivariable Cox proportional model of average TyG index quartiles, comparison of the lowest (Q1) and highest quartiles (Q4) indicated a significant positive association with the incidence of gout (HR: 1.326, 95% CI: 1.260–1.397). This association was non-linear (J-shape) when assessing the entire cohort and the diabetes and non-diabetes cohorts.

**Conclusion:**

Our study demonstrated that increased TyG index was associated with an incidence risk of gout in the general population. Additionally, this association was non-linear (J-shape) not only in the entire cohort, but also in diabetes mellitus and non-diabetes mellitus cohorts. The TyG index may be an important predictor of gout.

## 1 Introduction

Gout is a form of inflammatory arthritis characterized by recurrent attacks of red, tender, hot, and swollen joints. Pain typically onsets rapidly, reaching maximum intensity in less than 12 hours. The joint at the base of the big toe is affected in about 50% of gout patients (1). Gout is mainly associated with elevated uric acid concentrations in the blood. In hyperuricemia, monosodium urate crystals precipitate within joints, tendons, and surrounding tissues, eliciting an inflammatory response that may result in an episode of gout (2). Gout occurs more commonly in those who eat large amounts of meat, are heavy drinkers and/or smokers, and/or are overweight (2). Globally, the burden of gout is notable and is expected to increase. Therefore, novel methods to identify and correct modifiable factors that can increase the risk of gout are needed (3).

Insulin resistance is a common metabolic disorder that is often associated with type 2 diabetes mellitus (DM) (4). This condition occurs when the body’s cells become less responsive to insulin, a hormone essential for regulating blood sugar levels (5). The implications of insulin resistance extend beyond diabetes to include a wide range of health issues, such as hypertension, dyslipidemia, obesity, certain types of cancer, and liver, cardiovascular, neurodegenerative, inflammatory and infectious diseases (5–9). The triglyceride/high density lipoprotein (TG/HDL) ratio serves as a simple and practical surrogate marker for insulin resistance (10, 11). This index has gained recognition for its ease of use and cost-effectiveness, especially in settings where more direct and complex measurements of insulin resistance are not readily available. The TG/HDL ratio provides a valuable tool to assess metabolic health in various clinical settings and to identify individuals at risk of developing complications associated with insulin resistance (12–14).

Previous studies have suggested both correlations and causal links between increased insulin resistance and elevated serum uric acid concentations (15, 16). While hyperuricemia is an important risk factor for the onset of gout, not all patients with hyperuricemia develop gout. Therefore, further study of the association between insulin resistance and incidence risk of gout is needed. Moreover, few studies have assessed whether aggravation of insulin resistance may increase the incidence of gout. Additionally, the degree of insulin resistance may change over time, but few studies using repeated measured parameters in the general population have been completed. We hypothesize that elevated TG/HDL ratio is associated with development of gout. Our study aimed to investigate the association between the TG/HDL ratio and incidence of gout using longitudinal data obtained from the general population.

## 2 Materials and methods

### 2.1 Data source

The data for this study were obtained from the National Health Insurance Service-Health Screening Cohort (NHIS-HEALS) database, a subset of the Korean National Health Insurance Service (NHIS) database. The NHIS, a government program, provides health insurance to nearly 97% of the Korean population. The Medical Aid program, an affiliate of the NHIS, attends to the 3% of the population not covered by the NHIS. Our study was conducted based on the NHIS-HEALS cohort database of Republic of Korea (2002–2019) (17). The NHIS provides a nationwide free health screening program every two years for all Korean adults aged 40 and over.

The NHIS-HEALS database encompasses measurements of blood pressure, body mass index, blood biochemistry, a self-administered questionnaire addressing medical history, as well as lifestyle factors including smoking, alcohol consumption, and physical activity. Additionally, health claims data covering all participants’ hospital visits, diagnoses, surgeries, medical procedures, and prescriptions from 2002 to 2019 were included. Diagnoses at each hospital visit were recorded based on the International Classification of Disease, Tenth Revision (ICD-10). Demographic information such as sex, age, and household income were also included, and data regarding the participants’ health claims, insurance coverage maintenance, and death were available up to December 31, 2019.

### 2.2 Study population

**Figure 1:**
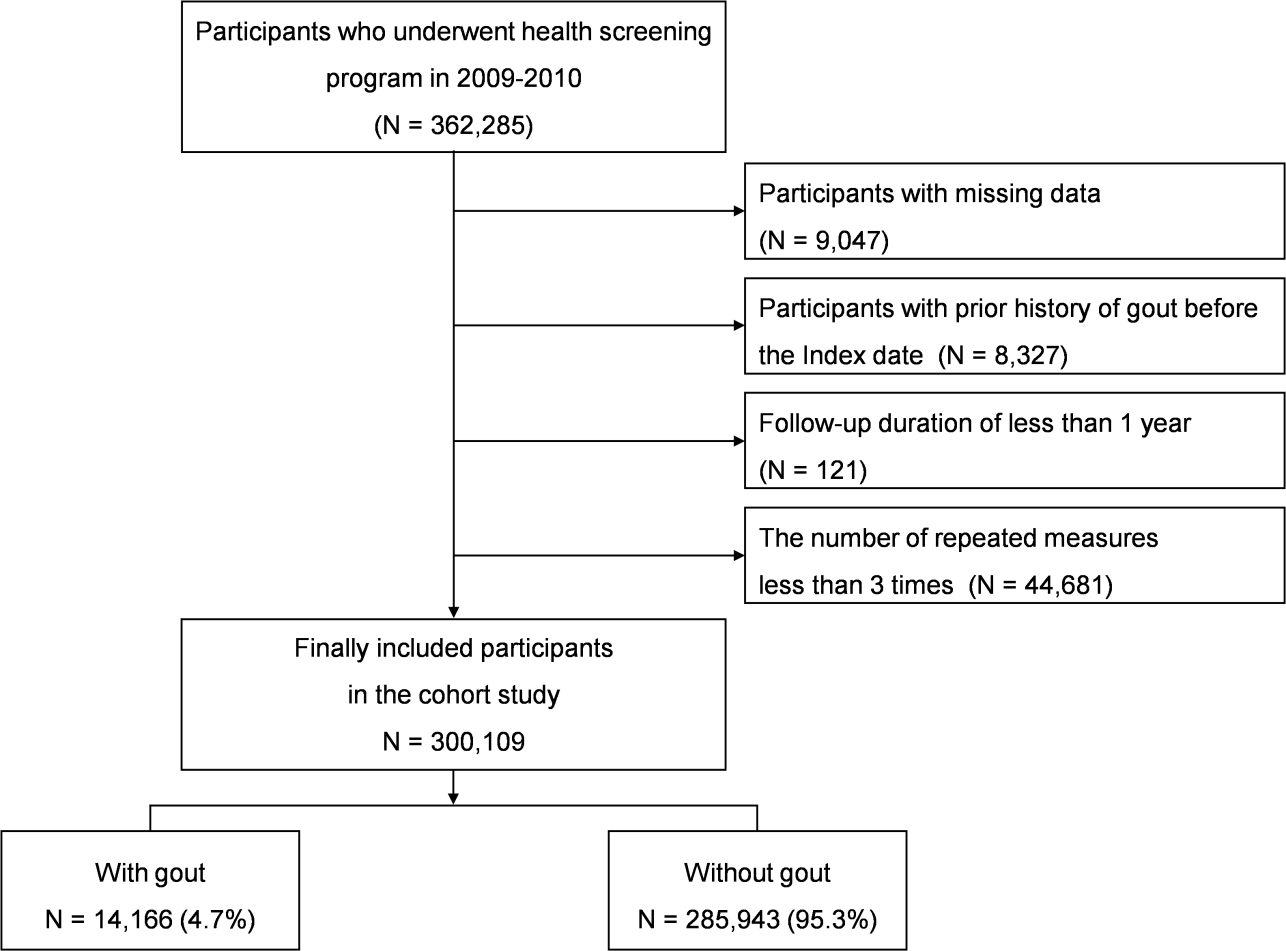
Flow chart of the patient inclusion and exclusion.

We included 362,285 participants from the NHIS-HEALS database who were aged 40 and over and participated in the national health screening program during the baseline years of 2009-2010. Among 362,285 participants, those with missing demographic, lifestyle, and/or laboratory data were excluded (n=9,047). The inclusion/exclusion period extended from 2002 up to the index date, during which patients with a history of gout were excluded (n=8,327). Participants with a follow-up duration less than one year (n=121) were excluded due to possible reverse causality or association, as were participants with fewer than three repeated measurements (n=44,681). After applying these inclusion and exclusion criteria, the final cohort for analysis comprised 300,109 individuals (**Figure 1**).

### 2.3 Data collection and definitions

Based on health claims data from the NHIS-HEALS, the participants’ demographic information (age, sex, body mass index (BMI; weight[kg]/height[m]2), waist circumference, household income) and lifestyle variables (smoking status, alcohol consumption, regular physical activity) were collected through self-reported questionnaires. Household income was categorized using quantiles of the individual’s health insurance premiums, with those in the 9th decile and above considered high income earners. Lifestyles were defined as follows: smoking status was categorized as never, former, or current smokers; the frequency of alcohol consumption was categorized as 0, 1–2, 3–4, or ≥5 drinks per week; the frequency of regular physical activity was categorized as 0, 1–4 days, or ≥5 days. Biochemical measurements included liver, lipid, and fasting glucose related blood laboratory findings. Hypertension, diabetes mellitus, dyslipidemia, renal disease, and liver disease were considered as comorbidities, and the Charlson comorbidity index (CCI) was used to assess the burden of covariates. Detailed definitions for these variables can be found in the **Supplementary Materials** (18–25).

### 2.4 TG/HDL ratio

In this study, the TG/HDL ratio was considered a time-dependent covariate throughout the follow-up period. Additional analyses used the average of at least three repeated measures of the TG/HDL ratio to reduce bias.

### 2.5 Outcome

Populations were identified as having gout based on at least two relevant claims, accompanied by prescriptions for at least one of febuxostat, allopurinol, colchicine, probenecid, steroid, and/or nonsteroidal anti-inflammatory drug, and with the initial date of diagnosis being noted. The diagnosis of gout was determined using the M10 code from the International Classification of Diseases, 10th Revision (ICD-10) (26). Follow-up was carried out until December 31, 2019, death, or the first occurrence of gout.

### 2.6 Statistical analysis

Comparisons between groups based on quartiles of the TG/HDL ratio were performed using a one-way ANOVA (analysis of variance) for continuous variables and the Chi-square test (or Fisher’s exact test) for categorical variables. Survival curves for the time-to-event outcomes were plotted using Kaplan-Meier curves, and the log-rank test was used to compare the survival curves across TG/HDL ratio quartile.

To evaluate the incidence of gout in relation to repeated measurements of the TG/HDL ratio during the follow-up period, the time-dependent Cox proportional hazards model was applied. For these analyses, the participants were divided into four quartiles (Q; Q1, Q2, Q3, and Q4) of the average TG/HDL ratio during the follow-up period. To ascertain the incidence of gout according to quartile, the conventional Cox proportional hazards model was utilized. The proportionality of the hazard assumption was evaluated using the Grambsch and Therneau test of Schoenfeld residuals, which yielded satisfactory results.

The results of time-dependent Cox regression and conventional Cox regression analyses were presented as hazard ratio (HR) and 95% confidence interval (CI) for an unadjusted model (Model 0), Model 1, and Model 2, depending on adjustment of covariates. Model 1 adjusted for age and sex, while Model 2 included Model 1 and additional adjustments for BMI, household income, smoking status, alcohol consumption, regular physical activity, hypertension, diabetes mellitus, renal disease, liver disease, and CCI. Blood biomarkers were not included as additional adjustments in multivariable Model 2 due to multi-collinearity because individuals with liver disease are more prone to exhibiting elevated liver enzymes and aberrant lipid metabolism (i.e., an elevated TG/HDL ratio) concurrently. Considering covariates, in cases where participants underwent multiple health check-ups from 2009 to 2019, the data from their latest examination were utilized for the statistical analyses. As insulin resistance is closely associated with diabetes mellitus, we performed sensitivity analyses according to presence of diabetes mellitus. To explore the relationship between the repeated measurements of average TG/HDL ratio and incidence of gout, restricted cubic splines were utilized. Subgroup analyses addressing the association of the TG/HDL ratio with gout were performed according to demographics, lifestyle, and covariates, with p-values suggesting interactions. All statistical analyses were conducted using SAS version 9.4 (SAS Inc., Cary, NC, USA) and R software, version 4.2.1 (R Foundation for Statistical Computing, Vienna, Austria), with statistical significance defined as a two-sided p-value <0.05.

### 2.7 Ethics approval and consent to participate

The ethical approval and participation consent protocols followed the Helsinki Declaration guidelines. Our institutional reviewer board approved our study (EUMC-2022-02-018). Given that the study data are accessible to the public through the NHIS database, the need for ethical approval and informed consent was waived.

## 3 Results

### 3.1 Baseline characteristics of participants

The numbers of measurements during the follow-up period are described in **Supplementary Table 1**, and the characteristics for each year are described in **Supplementary Table 2**.

**Table 1** presents the baseline characteristics of the entire cohort divided into quartiles of the average TG/HDL ratio (Q1 (<1.585), Q2 (1.585–2.300), Q3 (2.300–3.384), and Q4 (≥3.384)). The Q3 group was older than the other groups (Q1, Q2, and Q4). The Q4 group contained greater proportions of men and participants with obesity. On the other hand, the income level of the Q4 group was lower than in the other groups. Additionally, the Q4 group contained a greater proportion of participants who were current smokers and consumers of alcohol but who also engaged in fewer exercise sessions per week. With respect to the laboratory finding, including liver enzymes and TG, the Q4 group results were elevated in comparison to other groups, and proportions of comorbidities, including hypertension, diabetes mellitus, dyslipidemia, renal disease, and liver disease, and CCI scores of 2 or more were significantly greater in the Q4 group (**Table 1**).

**Table 1:** Baseline characteristics of study participants.

Variables	Total	TG/HDL ratio quartile				
		Q1 (< 1.585)	Q2 (1.585 - 2.300)	Q3 (2.300 - 3.384)	Q4 (≥ 3.384)	p-value
		Mean ± SD, N(%)	Mean ± SD, N(%)	Mean ± SD, N(%)	Mean ± SD, N(%)	
Number	300,109	75,028 (25.0)	75,026 (25.0)	75,027 (25.0)	75,028 (25.0)	
Age, years						<.001
< 65	237,745 (79.2)	61,771 (82.3)	58,400 (77.8)	57,898 (77.2)	59,676 (79.5)	
≥ 65	62,364 (20.8)	13,257 (17.7)	16,626 (22.2)	17,129 (22.8)	15,352 (20.5)	
Sex						<.001
Female	141,061 (47.0)	42,953 (57.2)	38,277 (51.0)	33,778 (45.0)	26,053 (34.7)	
Male	159,048 (53.0)	32,075 (42.8)	36,749 (49.0)	41,249 (55.0)	48,975 (65.3)	
Body mass index (kg/m^2^)						<.001
< 25	196,714 (65.5)	59,965 (79.9)	51,008 (68.0)	45,649 (60.8)	40,092 (53.4)	
≥ 25	103,395 (34.5)	15,063 (20.1)	24,018 (32.0)	29,378 (39.2)	34,936 (46.6)	
Waist circumference (cm)						<.001
Male < 90, female < 85	242,153 (80.7)	67,824 (90.4)	62,126 (82.8)	58,035 (77.4)	54,168 (72.2)	
Male ≥ 90, female ≥ 85	57,956 (19.3)	7,204 (9.6)	12,900 (17.2)	16,992 (22.6)	20,860 (27.8)	
Household income						0.007
Low	191,764 (63.9)	47,705 (63.6)	48,311 (64.4)	47,929 (63.9)	47,819 (63.7)	
High	108,345 (36.1)	27,323 (36.4)	26,715 (35.6)	27,098 (36.1)	27,209 (36.3)	
Smoking status						<.001
Never	194,919 (64.9)	56,080 (74.7)	51,484 (68.6)	47,318 (63.1)	40,037 (53.4)	
Former	55,623 (18.5)	11,851 (15.8)	13,227 (17.6)	14,530 (19.4)	16,015 (21.3)	
Current	49,567 (16.6)	7,097 (9.5)	10,315 (13.8)	13,179 (17.5)	18,976 (25.3)	
Alcohol consumption (days/week)						<.001
None	180,970 (60.3)	47,629 (63.5)	47,222 (62.9)	45,372 (60.5)	40,747 (54.3)	
1-2 times	78,784 (26.3)	18,661 (24.9)	18,527 (24.7)	19,698 (26.3)	21,898 (29.2)	
3-4 times	26,535 (8.8)	5,599 (7.5)	6,012 (8.0)	6,642 (8.9)	8,282 (11.0)	
≥ 5 times	13,820 (4.6)	3,139 (4.2)	3,265 (4.4)	3,315 (4.4)	4,101 (5.5)	
Regular physical activity (days/week)						<.001
None	74,196 (24.7)	17,266 (23.0)	18,676 (24.9)	19,117 (25.5)	19,137 (25.5)	
1-4 days	133,808 (44.6)	32,705 (43.6)	32,893 (43.8)	33,486 (44.6)	34,724 (46.3)	
≥ 5 days	92,105 (30.7)	25,057 (33.4)	23,457 (31.3)	22,424 (29.9)	21,167 (28.2)	
Laboratory findings						
AST (U/L)	26.2 ± 16.1	25.2 ± 16.2	25.6 ± 13.8	26.1 ± 16.1	27.7 ± 17.9	<.001
ALT (U/L)	25.0 ± 18.6	21.6 ± 17.2	23.8 ± 17.7	25.7 ± 18.2	29.0 ± 20.4	<.001
Total-C (mg/dL)	200.2 ± 37.1	195.2 ± 34.7	199.3 ± 36.4	202.2 ± 37.5	204.3 ± 38.9	<.001
HDL-C (mg/dL)	54.8 ± 23.7	65.3 ± 29.7	56.7 ± 23.8	51.4 ± 18.5	45.7 ± 15.8	<.001
LDL-C (mg/dL)	119.0 ± 35.7	115.7 ± 32.9	121.8 ± 34.5	123.0 ± 35.7	115.5 ± 38.6	<.001
Triglyceride (mg/dL)	136.4 ± 82.7	77.4 ± 32.8	108.2 ± 40.4	141.2 ± 52.9	218.8 ± 103.8	<.001
FBG (mg/dL)	100.5 ± 24.1	96.0 ± 19.0	99.0 ± 21.8	101.5 ± 24.4	105.3 ± 29.1	<.001
Comorbidities						
Hypertension	90,097 (30.0)	16,481 (22.0)	21,785 (29.0)	24,949 (33.3)	26,882 (35.8)	<.001
Diabetes mellitus	36,675 (12.2)	5,330 (7.1)	7,973 (10.6)	10,229 (13.6)	13,143 (17.5)	<.001
Dyslipidemia	47,249 (15.7)	8,728 (11.6)	11,327 (15.1)	13,057 (17.4)	14,137 (18.8)	<.001
Renal disease	38,678 (12.9)	8,469 (11.3)	9,408 (12.5)	10,041 (13.4)	10,760 (14.3)	<.001
Liver disease	49,344 (16.4)	10,932 (14.6)	11,974 (16.0)	12,827 (17.1)	13,611 (18.1)	<.001
Charlson comorbidity index						<.001
0	156,677 (52.2)	41,272 (55.0)	39,048 (52.0)	37,930 (50.6)	38,427 (51.2)	
1	123,739 (41.2)	29,154 (38.9)	31,144 (41.5)	32,099 (42.7)	31,342 (41.8)	
2 or more	19,693 (6.6)	4,602 (6.1)	4,834 (6.5)	4,998 (6.7)	5,259 (7.0)	

TG, triglyceride; HDL, high-density lipoprotein; Q, quartile; SD, standard deviation; N, number; AST, aspartate aminotransferase; ALT, alanine aminotransferase; Total-C, total cholesterol; HDL-C, high-density lipoprotein cholesterol; LDL-C, low-density lipoprotein cholesterol; FBS, fasting blood glucose.

### 3.2 Relationship of the TG/HDL ratio with incidence of gout

**Figure 2:**
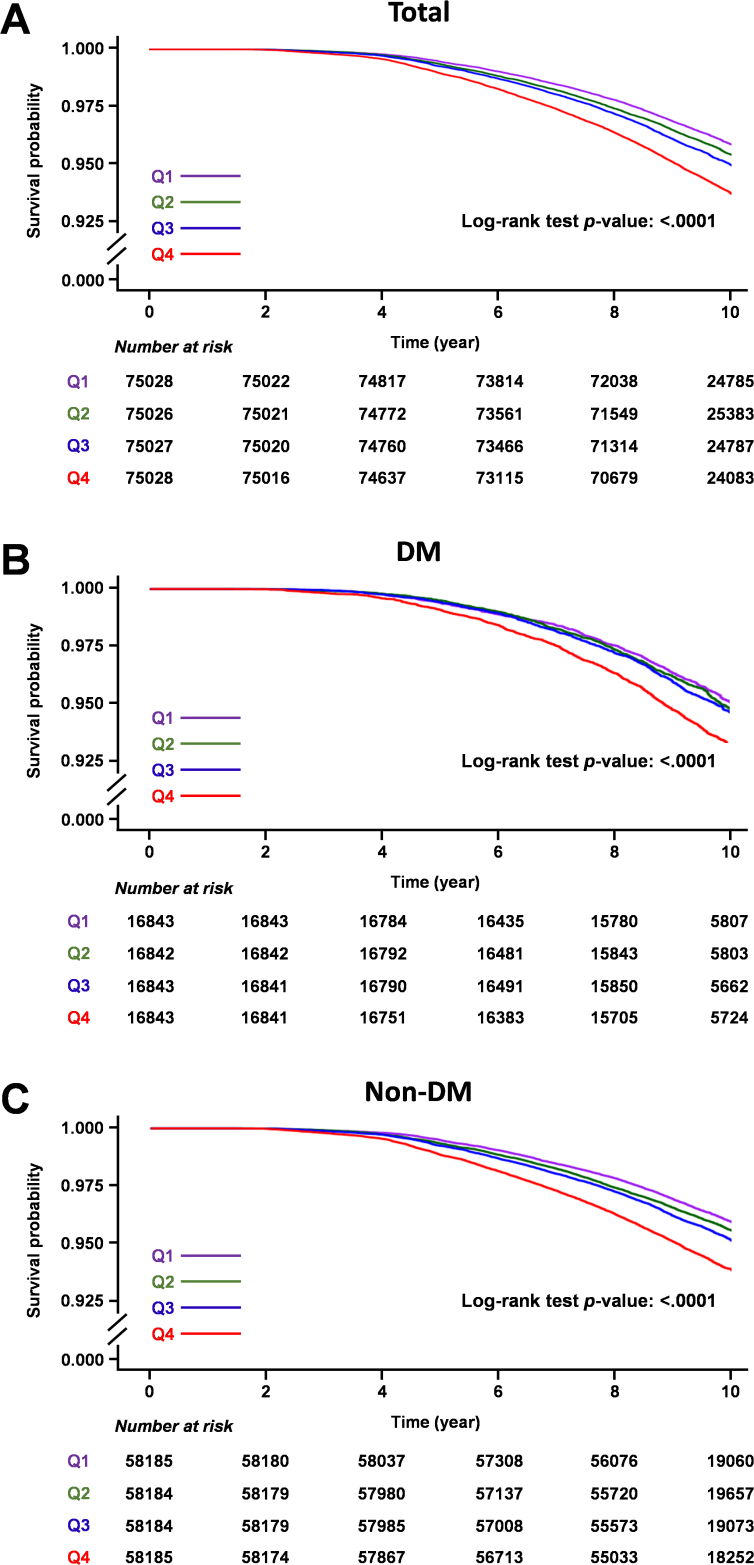
Kaplan-Meier survival curves of gout outcomes according to TG/HDL ratio quartiles. **(A)** Total cohort, **(B)** Diabetic mellitus cohort, **(C)** Non-diabetic mellitus cohort.

During the median 9.62 years of follow-up (interquartile range 8.72–10.53), 14,116 individuals (4.70%) developed gout. Survival curves depicting the incidence of gout across quartiles of average TG/HDL ratio are presented in **Figure 2**. Higher TG/HDL ratio quartiles (Q1 to Q4) were associated with an increased risk of gout (log-rank test for the entire cohort: p <0.001, DM cohort: p <0.001, and non-DM cohort: p <0.001).

Considering the results of the multivariable time-dependent Cox proportional hazards model using the repeated measurements of the TG/HDL ratio, a unit increase in TG/HDL ratio significantly increased the risk of gout in the entire cohort (HR: 1.014, 95% CI: 1.011 - 1.017), DM cohort (HR: 1.018. 95% CI: 1.008 - 1.028), and non-DM cohort (HR: 1.014, 95% CI: 1.011 - 1.017) in fully adjusted multivariable models (**Table 2**, **Supplementary Table 3**).

**Table 2:** Results of risk of gout considering the TG/HDL ratio as a time-dependent covariate.

Groups	N	Events	Person-years	Incidence rate (per 1000 person-years)	Unadjusted	Model 1	Model 2
					HR (95% CI)	HR (95% CI)	HR (95% CI)
Total	300,109	14,166	2,877,865	4.922	1.019 (1.017, 1.022)	1.018 (1.015, 1.020)	1.014 (1.011, 1.017)
DM	67,371	3,477	644,691	5.393	1.025 (1.017, 1.033)	1.019 (1.011, 1.028)	1.018 (1.008, 1.028)
Non-DM	232,738	10,689	2,233,174	4.786	1.019 (1.016, 1.021)	1.017 (1.014, 1.020)	1.014 (1.011, 1.017)

TG, triglyceride; HDL, high-density lipoprotein; N, number; HR, hazard ratio; CI, confidence interval; DM, diabetes mellitus. The estimated HR (95% CI) was calculated using time-dependent Cox regression model. Model 1 was adjusted for age and sex. Model 2 was adjusted for age, sex, body mass index, household income, smoking status, alcohol consumption, regular physical activity, hypertension, diabetes mellitus, dyslipidemia, renal disease, liver disease and Charlson comorbidity index.

**Figure 3:**
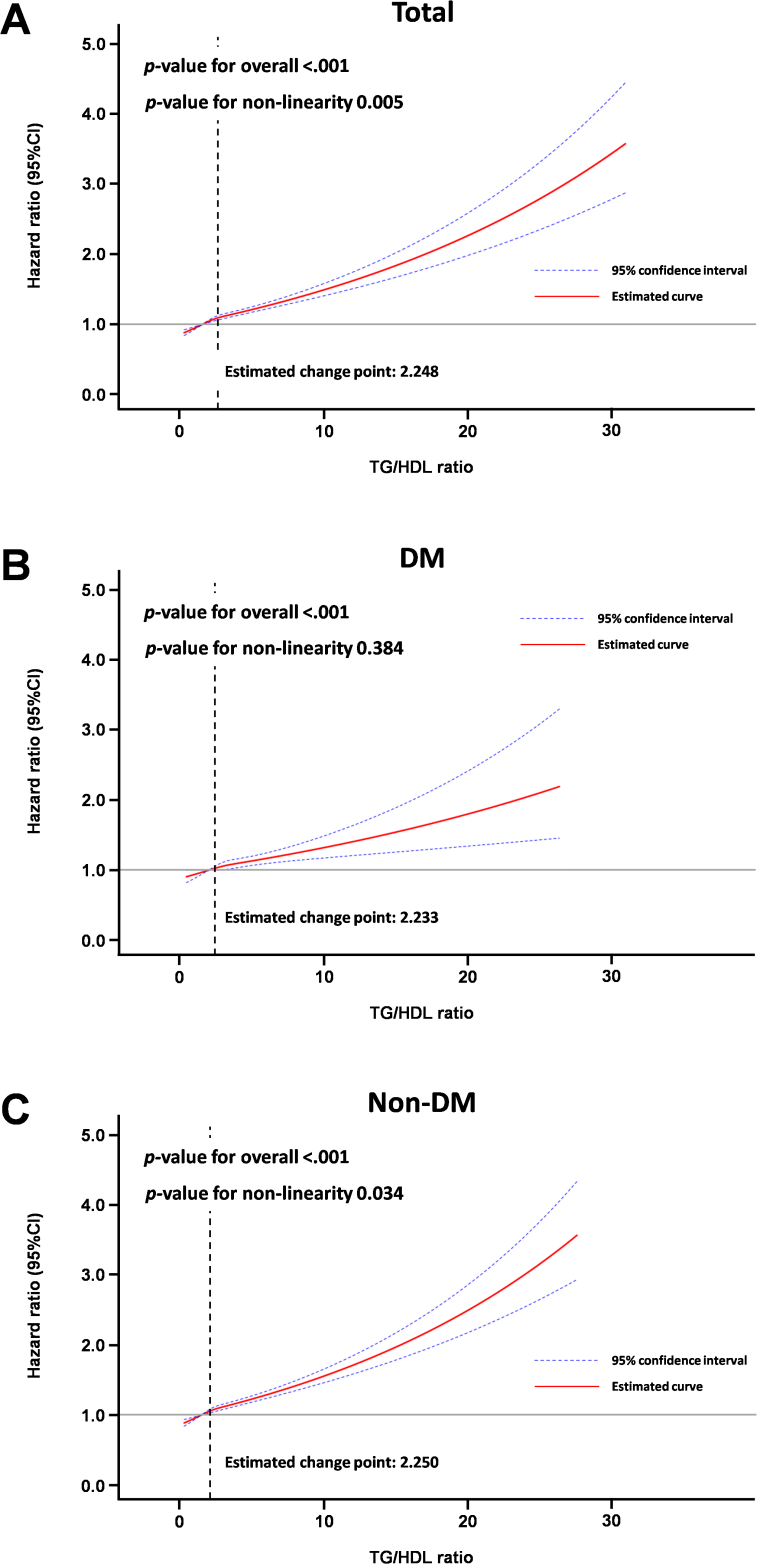
Spline curve for incidence of gout according to average TG/HDL ratio. **(A)** Total cohort, **(B)** Diabetic mellitus cohort, **(C)** Non-diabetes mellitus cohort.

Results of multivariable Cox proportional model for average TG/HDL ratio quartiles during follow-up are detailed in **Table 3** and **Supplementary Table 4**. Compared with the lowest quartile (Q1), the highest quartile (Q4) was significantly positively associated with incidence of gout (HR: 1.299, 95% CI: 1.236–1.366 for the entire cohort; HR: 1.253, 95% CI: 1.138–1.379 for the DM cohort; HR: 1.305, 95% CI: 1.234–1.381 for the non-DM cohort) in fully adjusted multivariable analyses. Moreover, on visual inspection, the restricted cubic spline analysis displayed a pattern of positive association of TG/HDL ratio with incidence of gout (**Figure 3**).

**Table 3:** Risk of gout based on the average TG/HDL ratio quartile during the follow-up period.

Average of repeated measured TG/HDL ratio	N	Events	Person-years	Incidence rate (per 1000 person-years)	Unadjusted	Model 1	Model 2
					HR (95% CI)	HR (95% CI)	HR (95% CI)
Total							
Q1 (< 1.585)	75,028	2,914	721,118	4.041	ref	ref	ref
Q2 (1.585 - 2.300)	75,026	3,245	721,109	4.500	1.112 (1.058, 1.169)	1.125 (1.070, 1.183)	1.068 (1.016, 1.124)
Q3 (2.300 - 3.384)	75,027	3,564	719,678	4.952	1.226 (1.168, 1.288)	1.225 (1.166, 1.287)	1.120 (1.065, 1.178)
Q4 (≥ 3.384)	75,028	4,443	715,960	6.206	1.544 (1.474, 1.618)	1.467 (1.399, 1.538)	1.299 (1.236, 1.366)
p-value for trend					<.0001	<.0001	<.0001
DM							
Q1 (< 1.980)	16,843	750	161,117	4.655	ref	ref	ref
Q2 (1.980 - 2.832)	16,842	795	161,535	4.922	1.054 (0.954, 1.165)	1.047 (0.948, 1.157)	1.016 (0.919, 1.123)
Q3 (2.832 - 4.095)	16,843	851	161,423	5.272	1.131 (1.025, 1.248)	1.094 (0.992, 1.207)	1.038 (0.940, 1.146)
Q4 (≥ 4.095)	16,843	1,081	160,617	6.730	1.450 (1.321, 1.592)	1.334 (1.214, 1.465)	1.253 (1.138, 1.379)
p-value for trend					<.0001	<.0001	<.0001
Non-DM							
Q1 (< 1.503)	58,185	2,218	559,646	3.963	ref	ref	ref
Q2 (1.503 - 2.165)	58,184	2,435	559,757	4.350	1.096 (1.035, 1.161)	1.106 (1.044, 1.171)	1.054 (0.994, 1.116)
Q3 (2.165 - 3.165)	58,184	2,664	558,507	4.770	1.204 (1.138, 1.274)	1.198 (1.132, 1.268)	1.104 (1.042, 1.169)
Q4 (≥ 3.165)	58,185	3,372	555,264	6.073	1.541 (1.461, 1.626)	1.456 (1.379, 1.538)	1.305 (1.234, 1.381)
p-value for trend					<.0001	<.0001	<.0001

TG, triglyceride; HDL, high-density lipoprotein; N, number; HR, hazard ratio; CI, confidence interval; Q, quartile; DM, diabetes mellitus. The estimated HR (95% CI) was derived from the conventional Cox regression model. Model 1 was adjusted for age and sex. Model 2 was adjusted for age, sex, body mass index, household income, smoking status, alcohol consumption, regular physical activity, hypertension, diabetes mellitus, dyslipidemia, renal disease, liver disease, and Charlson comorbidity index.

### 3.3 Subgroup analyses for association of TG/HDL ratio with incidence of gout

**Figure 4:**
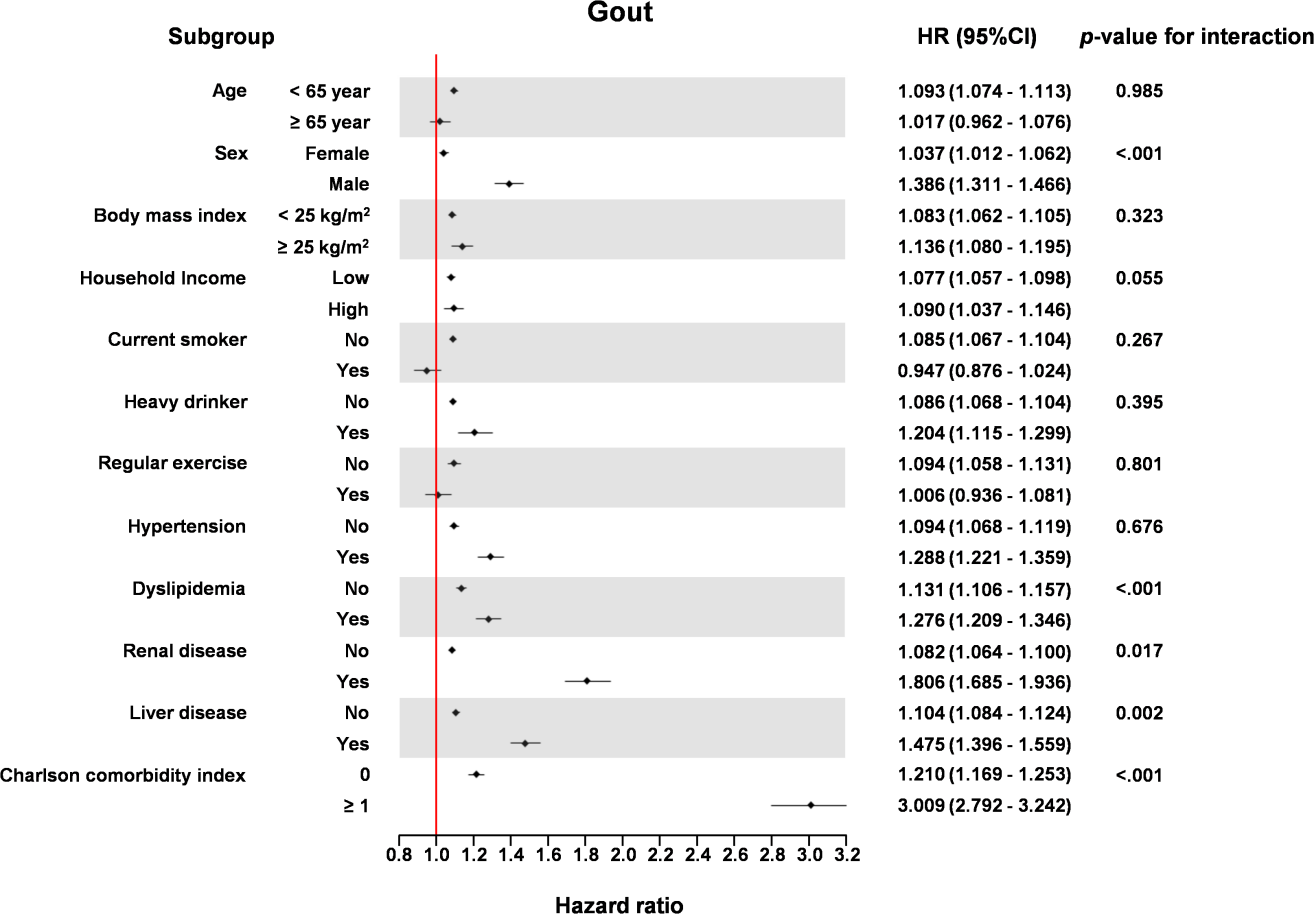
Forest plots of incidence of gout according to the demographic and comorbidity data.

The association of TG/HDL ratio with HF incidence was significant in the older age group (≥65 years) compared to the younger age group (<65 years) (P for interaction = 0.004), in men compared to women (P for interaction < 0.001), in patients with dyslipidemia compared to those without dyslipidemia (P for interaction = 0.004), in patients with renal disease compared to those without renal disease (P for interaction = 0.002), and in patients with liver disease compared to those without liver disease (P for interaction = 0.002) (**Figure 4**).

## 4 Discussion

The key findings of our study were that the TG/HDL ratio was associated with incidence risk of gout in the general population based on time-dependent analyses of the TG/HDL ratio and conventional Cox regression analyses of the average value of repeated measures of the TG/HDL ratio.

The TG/HDL ratio is linked to the presence and progression of several health conditions. For example, an increased TG/HDL ratio has been correlated with greater risk of metabolic syndrome, cerebrovascular disease, coronary artery disease, and peripheral arterial disease (27). Notably, in patients with coronavirus disease 2019 (COVID19), elevated TG/HDL ratios have been associated with more severe illness and increased mortality rates (28). Moreover, a previous study showed a significant positive association between the TG/HDL ratio and long-term all-cause mortality in patients with coronary artery disease (29). In the China Health and Retirement Longitudinal Study, baseline TG/HDL ratios were associated with hyperuricemia (30). In a cross-sectional study of US National Health and Nutrition Examination Survey data, hyperuricemia was positively associated with elevation of TG/HDL ratio (31). Nevertheless, the above-noted studies did not investigate the association of the TG/HDL ratio with incidence of gout. Our longitudinal study is meaningful in that it presents additional information regarding the association between repeated measures of the TG/HDL ratio and incidence of gout using a large sample of the general population.

In subgroup analysis, both men and women showed positive association with TG/HDL ratio and incidence of gout, while men had higher association than women. This difference in sex might have been caused by protective effect of estrogen in the incidence of gout. However, this result should be interpreted with caution, and further analysis about sex difference in the occurrence of gout with insulin resistance is in need. The presence of dyslipidemia is often associated with elevated TG levels, which in turn may result in a higher TG/HDL ratio, potentially strengthening its association with gout development. Renal disease is an independent risk factor for gout, and when combined with an elevated TG/HDL ratio, the risk of gout may be further amplified. Liver disease increases the likelihood of hyperlipidemia, which can contribute to higher TG levels, thereby necessitating careful monitoring. The CCI, as a measure of multiple comorbidities, should be interpreted cautiously since the presence of numerous comorbidities may significantly heighten the risk of gout.

Our study could not establish the underlying mechanism of this association. Future research could benefit from integrating biochemical or genetic data to explore these pathways. Although our study was not mechanistic, there are several plausible hypotheses regarding the association between TG/HDL ratio and incidence risk of gout. The TG/HDL ratio, derived from fasting plasma glucose and TG concentrations, is considered an easy and dependable alternate clinical indicator for metabolic syndrome and insulin resistance (32). Consequently, the link between the TG/HDL ratio and gout may be due to a mechanism involving insulin resistance. In individuals with insulin resistance, glycolysis intermediates are converted into 5-phosphoribose and ribose phosphate pyrophosphate, resulting in a boost in serum uric acid production (33). Furthermore, elevated insulin concentrations due to insulin resistance promote the Na+-H+ exchange in the renal tubules, leading to enhanced H+ elimination and increased reabsorption of uric acid (34). Additionally, activation of the renin-angiotensin system triggered by hyperinsulinemia reduces renal blood flow and augments urate reabsorption. This process also generates xanthine oxidase, subsequently escalating uric acid production (35). Increased blood uric acid concentrations may enhance risk of gout. Furthermore, long-term dyslipidemia may lead to stenosis or occlusion of the small arteries in the kidneys, eventually leading to disorders of urate excretion. A previous study showed that serum TG concentrations were an independent and significant risk factor for elevated serum uric acid concentrations (36).

We acknowledge some limitations of our study. First, our findings may not be generalizable to different ethnic groups, as our study enrolled exclusively Korean participants. Additional research is needed because our results may differ across races and cultures. Second, despite multiple assessments of the TG/HDL ratio to enhance reliability, the retrospective nature of the study limits the establishment of a causal relationship. Third, the reliance on health screening data from the general populace means some key gout-related biomarkers, like serum uric acid concentrations, were not included in the data obtained. Our study did not include potential confounders such as dietary intake and medication use which can influence both the TG/HDL ratio and gout incidence.

## 5 Conclusion

Our study demonstrated that increased TG/HDL ratios were associated with the incidence of gout in the general population regardless of accompanying diabetes mellitus. The TG/HDL ratio may be an important tool to predict the incidence of gout in the general population. Future research is needed to explore causality and investigate interventions targeting the TG/HDL ratio and gout.

## Data Availability

Publicly available datasets were analyzed in this study. This data can be found here: http://nhiss.nhis.or.kr/bd/ab/bdaba021eng.do, the National Health Insurance Service—National Health Screening Cohort (NHIS-HEALS) database.
