# Supplementary material for: Association between the triglyceride/high-density lipoprotein (TG/HDL) ratio and incidence of gout: A nationwide cohort study
Source: Front Endocrinol (Lausanne). 2025 Jan 10;15:1453458. doi: 10.3389/fendo.2024.1453458 (PMC11757121; doi:10.3389/fendo.2024.1453458)
Supplement: Supplementary file 1 [file DataSheet1.docx]

Supplementary Material

# Supplementary methods

Definition of covariates

Smoking history (never, former, and current)

Definition and measurement of variables like usual smoking habits were obtained by questionnaire in the health examination program. Smoking status was used to categorize participants into three groups: none, former smoker, and current smoker. Current smoker was classified according to the WHO definition as a person who has smoked more than five packs (100 cigarettes) in a lifetime and smoked daily or occasionally for the last 28 days. Former smoker was defined as a person who had smoked more than 100 cigarettes in a lifetime and had not smoked in the last 28 days.

Alcohol consumption (none, moderate, and heavy)

Definition and measurement of variables like usual alcohol consumption were obtained by questionnaire in the health examination program. Alcohol consumption was categorized into three groups: none, moderate drinker, and heavy drinker. Differentiation between moderate and heavy drinker was based on whether a patient usually takes more than 14 drinks/7 drinks per week for men/women. The drinks were calculated by multiplying the average drinking frequency per week by the number of drinks per occasion.

Physical activity (low, moderate)

Physical activity was assessed using the Korean version of the International Physical Activity Questionnaire-short form. We created composite physical activity based on Metabolic Equivalent Task (MET)-minutes/week (walking: 3.3 METs; moderate physical activity: 4.0 METs; vigorous physical activity: 8.0 METs), which was categorized as follows based on total physical activity metabolic equivalents: low (< 600 METs), moderate (600–2,999 METs), and vigorous (≥ 3,000 METs).

Comorbidities

Hypertension

Hypertension was defined as using at least one claim of ICD-10 code(I10-15) with the prescription of an anti-hypertensive agent, claims of ICD-10 code(I10-15) more than two times, a systolic blood pressure of ≥140 mmHg and a diastolic blood pressure of ≥90 mmHg or positive checking in self-report questionnaire on hypertension in the health examination program.

Diabetes mellitus

Diabetes mellitus was as defined using at least one claim of ICD-10 code(E11-14) with the prescription of an anti-diabetic agent, claims of ICD-10 code(E11-14) more than two times, fasting serum glucose concentration of ≥7.0 mmol/L or positive checking in self-report questionnaire on diabetes mellitus in the health examination program.

Dyslipidemia

Dyslipidemia was defined as using at least one claim of ICD-10 code(E78) with the prescription of an anti-dyslipidemic agent, claims of ICD-10 code(E78) more than two times or total cholesterol level of ≥240 mg/dL.

Renal disease

Renal disease was defined as using claims of ICD–10 codes (N17-19, I12-13, E08.2, E10.2, E11.2, E13.2) more than two times or estimated glomerular filtration rate (eGFR) of <60 mL/min/1.73m2.

Liver disease

Liver disease was defined as using claims of ICD-10 codes (B18, K70, K71, K72, K73, K74, K76.1) with more than two times of admission or outpatient department.

Charlson comorbidity index (0, 1, or ≥2).

The Charlson comorbidity index score was calculated for each subject based on diseases diagnosed before index date and divided into three groups (0, 1, and ≥2 scores).

# Supplementary Tables

**Supplementary Table 1.** The number of health screenings conducted during the follow-up period.

| Number of  measurements | Total (N=310,109) | DM (N=67,371) | Non-DM (N=232,738) |
| --- | --- | --- | --- |
| 3 | 42,979 (14.32%) | 11,277 (16.74%) | 31,702 (13.62%) |
| 4 | 78,441 (26.14%) | 18,642 (27.67%) | 59,799 (25.69%) |
| 5 | 81,373 (27.11%) | 17,793 (26.41%) | 63,580 (27.32%) |
| 6 | 39,414 (13.13%) | 8,379 (12.43%) | 31,035 (13.35%) |
| 7 | 21,152 (7.06%) | 4,385 (6.51%) | 16,767 (7.20%) |
| 8 | 23,680 (7.89%) | 4,445 (6.60%) | 19,235 (8.26%) |
| 9 | 12,726 (4.24%) | 2,371 (3.52%) | 10,355 (4.45%) |
| 10 | 344 (0.11%) | 79 (0.12%) | 265 (0.11%) |

Abbreviations: N, number; DM, diabetes mellitus.

**Supplementary Table 2.** Temporal Changes of variables throughout the follow-up period.

| Variables | 2009, (N=214,281) | 2010, (N=219,219) | 2011, (N=218,889) | 2012, (N=211,229) | 2013, (N=200,230) | 2014, (N=204,434) | 2015, (N=216,149) | 2016, (N=199,799) | 2017, (N=207,233) | 2018, (N=199,597) | 2019, (N=208,172) |
| --- | --- | --- | --- | --- | --- | --- | --- | --- | --- | --- | --- |
| Age, years |  |  |  |  |  |  |  |  |  |  |  |
| < 65 | 167,872 (78.3) | 173,877 (79.3) | 164,953 (75.4) | 161,489 (76.5) | 142,969 (71.4) | 147,631 (72.2) | 145,590 (67.4) | 126,060 (63.1) | 120,181 (58.0) | 112,294 (56.3) | 105,713 (50.8) |
| ≥ 65 | 46,409 (21.7) | 45,342 (20.7) | 53,936 (24.6) | 49,740 (23.5) | 57,261 (28.6) | 56,803 (27.8) | 70,559 (32.6) | 73,739 (36.9) | 87,052 (42.0) | 87,303 (43.7) | 102,459 (49.2) |
| Sex, N (%) |  |  |  |  |  |  |  |  |  |  |  |
| Female | 94,738 (44.2) | 91,021 (41.5) | 96,971 (44.3) | 88,145 (41.7) | 89,304 (44.6) | 86,162 (42.1) | 95,148 (44.0) | 85,635 (42.9) | 92,959 (44.9) | 86,352 (43.3) | 94,930 (45.6) |
| Male | 119,543 (55.8) | 128,198 (58.5) | 121,918 (55.7) | 123,084 (58.3) | 110,926 (55.4) | 118,272 (57.9) | 121,001 (56.0) | 114,164 (57.1) | 114,274 (55.1) | 113,245 (56.7) | 113,242 (54.4) |
| Body mass index (kg/m2) |  |  |  |  |  |  |  |  |  |  |  |
| < 25 | 140,192 (65.4) | 143,894 (65.6) | 143,365 (65.5) | 138,853 (65.7) | 131,619 (65.7) | 133,713 (65.4) | 138,788 (64.2) | 125,578 (62.9) | 128,158 (61.8) | 122,069 (61.2) | 127,494 (61.2) |
| ≥ 25 | 74,089 (34.6) | 75,325 (34.4) | 75,524 (34.5) | 72,376 (34.3) | 68,611 (34.3) | 70,721 (34.6) | 77,361 (35.8) | 74,221 (37.1) | 79,075 (38.2) | 77,528 (38.8) | 80,678 (38.8) |
| Waist circumference (cm) |  |  |  |  |  |  |  |  |  |  |  |
| Male < 90, female < 85 | 163,426 (76.3) | 168,698 (77.0) | 167,089 (76.3) | 162,119 (76.8) | 151,692 (75.8) | 155,021 (75.8) | 159,504 (73.8) | 145,682 (72.9) | 149,367 (72.1) | 143,061 (71.7) | 146,731 (70.5) |
| Male ≥ 90, female ≥ 85 | 50,855 (23.7) | 50,521 (23.0) | 51,800 (23.7) | 49,110 (23.2) | 48,538 (24.2) | 49,413 (24.2) | 56,645 (26.2) | 54,117 (27.1) | 57,866 (27.9) | 56,536 (28.3) | 61,441 (29.5) |
| Household income, N (%) |  |  |  |  |  |  |  |  |  |  |  |
| Low | 144,161 (67.3) | 137,480 (62.7) | 148,053 (67.6) | 130,303 (61.7) | 132,974 (66.4) | 130,163 (63.7) | 143,052 (66.2) | 132,528 (66.3) | 138,966 (67.1) | 134,153 (67.2) | 140,175 (67.3) |
| High | 70,120 (32.7) | 81,739 (37.3) | 70,836 (32.4) | 80,926 (38.3) | 67,256 (33.6) | 74,271 (36.3) | 73,097 (33.8) | 67,271 (33.7) | 68,267 (32.9) | 65,444 (32.8) | 67,997 (32.7) |
| Smoking status, N (%) |  |  |  |  |  |  |  |  |  |  |  |
| Never | 135,217 (63.1) | 133,805 (61.0) | 136,742 (62.5) | 128,487 (60.8) | 125,693 (62.8) | 125,494 (61.4) | 136,207 (63.0) | 123,939 (62.0) | 132,212 (63.8) | 124279 (62.3) | 128964 (62.0) |
| Former | 39,588 (18.5) | 45,053 (20.6) | 45,105 (20.6) | 46,557 (22.0) | 42,836 (21.4) | 46,950 (23.0) | 51,999 (24.1) | 49,036 (24.5) | 50,231 (24.2) | 48275 (24.2) | 50962 (24.5) |
| Current | 39,476 (18.4) | 40,361 (18.4) | 37,042 (16.9) | 36,185 (17.2) | 31,701 (15.8) | 31,990 (15.6) | 27,943 (12.9) | 26,824 (13.5) | 24,790 (12.0) | 26930 (13.5) | 28081 (13.5) |
| Alcohol consumption (days/week), N (%) |  |  |  |  |  |  |  |  |  |  |  |
| None | 126,066 (58.8) | 126,771 (57.8) | 131,606 (60.1) | 123,923 (58.7) | 122,946 (61.4) | 122,277 (59.8) | 133,240 (61.6) | 123,438 (61.8) | 132,348 (63.9) | 128069 (64.2) | 133749 (64.3) |
| 1-2 times | 57,930 (27.0) | 61,311 (28.0) | 56,583 (25.8) | 57,921 (27.4) | 50,448 (25.2) | 54,700 (26.8) | 54,590 (25.3) | 50,559 (25.3) | 49,692 (24.0) | 47477 (23.8) | 49090 (23.6) |
| 3-4 times | 19,721 (9.3) | 20,899 (9.5) | 20,041 (9.2) | 19,731 (9.3) | 17,579 (8.8) | 18,332 (9.0) | 18,821 (8.7) | 17,161 (8.6) | 16,462 (7.9) | 15360 (7.7) | 16433 (7.9) |
| ≥ 5 times | 10,564 (4.9) | 10,238 (4.7) | 10,659 (4.9) | 9,654 (4.6) | 9,257 (4.6) | 9,125 (4.4) | 9,498 (4.4) | 8,641 (4.3) | 8,731 (4.2) | 8578 (4.3) | 8736 (4.2) |
| Regular physical activity (days/week) , N (%) |  |  |  |  |  |  |  |  |  |  |  |
| None | 55,403 (25.9) | 52,208 (23.8) | 53,261 (24.4) | 47,736 (22.6) | 46,095 (23.0) | 42,353 (20.7) | 44,617 (20.6) | 39,040 (19.5) | 39,083 (18.9) | 36306 (18.2) | 37649 (18.1) |
| 1-4 days | 94,882 (44.2) | 99,827 (45.6) | 95,711 (43.7) | 96,328 (45.6) | 88,190 (44.1) | 93,408 (45.7) | 95,668 (44.3) | 88,226 (44.2) | 89,324 (43.1) | 86576 (43.4) | 89859 (43.2) |
| ≥ 5 days | 63,996 (29.9) | 67,184 (30.6) | 69,917 (31.9) | 67,165 (31.8) | 65,945 (32.9) | 68,673 (33.6) | 75,864 (35.1) | 72,533 (36.3) | 78,826 (38.0) | 76602 (38.4) | 80499 (38.7) |
| Laboratory findings |  |  |  |  |  |  |  |  |  |  |  |
| AST (U/L) | 26.5 ± 15.7 | 26.4 ± 16.8 | 26.4 ± 14.8 | 26.7 ± 17.6 | 26.6 ± 15.9 | 26.7 ± 16.2 | 27.1 ± 15.7 | 27.0 ± 15.9 | 27.4 ± 17.0 | 27.6 ± 18.5 | 27.8 ± 27.6 |
| ALT (U/L) | 25.3 ± 19.2 | 25.2 ± 18.4 | 24.5 ± 17.9 | 24.9 ± 19.3 | 24.6 ± 18.2 | 24.9 ± 18.5 | 25.1 ± 18.5 | 25.1 ± 18.6 | 25.0 ± 19.8 | 25.1 ± 20.2 | 24.8 ± 20.7 |
| Total-C (mg/dL) | 199.9 ± 37.4 | 199.3 ± 37.0 | 198.0 ± 37.3 | 197.7 ± 37.2 | 197.1 ± 37.7 | 195.4 ± 37.9 | 194.7 ± 38.8 | 194.5 ± 39.4 | 192.4 ± 39.8 | 190.8 ± 40.3 | 187.8 ± 40.7 |
| HDL-C (mg/dL) | 55.1 ± 29.0 | 54.0 ± 17.4 | 54.1 ± 15.6 | 54.1 ± 17.2 | 53.7 ± 15.3 | 53.6 ± 14.4 | 54.1 ± 14.5 | 54.4 ± 15.3 | 54.8 ± 14.2 | 55.1 ± 17.4 | 55.2 ± 14.4 |
| LDL-C (mg/dL) | 118.1 ± 36.6 | 118.3 ± 35.1 | 117.3 ± 34.6 | 117.3 ± 34.6 | 116.9 ± 34.9 | 115.8 ± 35.2 | 114.9 ± 35.4 | 114.4 ± 36.1 | 111.7 ± 36.3 | 110.2 ± 36.9 | 107.8 ± 36.9 |
| Triglyceride (mg/dL) | 140.4 ± 89.1 | 136.6 ± 80.4 | 134.2 ± 78.2 | 133.7 ± 80.4 | 133.7 ± 80.1 | 132.2 ± 79.4 | 130.7 ± 78.6 | 130.8 ± 78.4 | 130.7 ± 78.1 | 130.1 ± 77.5 | 124.9 ± 71.3 |
| FBG (mg/dL) | 100.8 ± 25.4 | 101.1 ± 25.1 | 101.3 ± 24.8 | 101.7 ± 24.9 | 102.6 ± 25.4 | 103.3 ± 25.5 | 103.9 ± 25.6 | 104.6 ± 25.7 | 105.0 ± 25.8 | 105.5 ± 25.4 | 105.9 ± 25.3 |
| Comorbidities |  |  |  |  |  |  |  |  |  |  |  |
| Hypertension, N (%) | 81,422 (38.0) | 82,924 (37.8) | 88,319 (40.3) | 85,497 (40.5) | 85,650 (42.8) | 86,499 (42.3) | 95,238 (44.1) | 79,274 (39.7) | 86,142 (41.6) | 85,141 (42.7) | 92,755 (44.6) |
| Diabetes mellitus, N (%) | 30,136 (14.1) | 30,796 (14.0) | 33,180 (15.2) | 32,505 (15.4) | 33,411 (16.7) | 34,748 (17.0) | 38,897 (18.0) | 36,201 (18.1) | 39,659 (19.1) | 39,876 (20.0) | 43,651 (21.0) |
| Dyslipidemia, N (%) | 58,915 (27.5) | 62,831 (28.7) | 68,438 (31.3) | 70,094 (33.2) | 73,659 (36.8) | 78,236 (38.3) | 91,505 (42.3) | 91,421 (45.8) | 101,695 (49.1) | 93,967 (47.1) | 106,467 (51.2) |
| Renal disease, N (%) | 32,061 (15.0) | 28,981 (13.2) | 26,591 (12.1) | 22,743 (10.8) | 23,215 (11.6) | 21,989 (10.8) | 23,664 (10.9) | 21,343 (10.7) | 23,259 (11.2) | 22,914 (11.5) | 25,245 (12.1) |
| Liver disease, N (%) | 34,654 (16.2) | 36,651 (16.7) | 39,370 (18.0) | 39,989 (18.9) | 41,130 (20.5) | 42,479 (20.8) | 48,841 (22.6) | 48,409 (24.2) | 53,458 (25.8) | 58,227 (29.2) | 67,838 (32.6) |
| Charlson comorbidity index, N (%) |  |  |  |  |  |  |  |  |  |  |  |
| 0 | 213,991 (99.9) | 218,927 (99.9) | 218,650 (99.9) | 211,020 (99.9) | 200,076 (99.9) | 204,232 (99.9) | 215,994 (99.9) | 199,661 (99.9) | 207,080 (99.9) | 199,479 (99.9) | 208,073 (99.9) |
| 1 | 246 (0.1) | 247 (0.1) | 200 (0.1) | 170 (0.1) | 131 (0.1) | 171 (0.1) | 132 (0.1) | 116 (0.1) | 126 (0.1) | 107 (0.1) | 78 (0.1) |
| 2 or more | 44 (0.0) | 45 (0.0) | 39 (0.0) | 39 (0.0) | 23 (0.0) | 31 (0.0) | 23 (0.0) | 22 (0.0) | 27 (0.1) | 11 (0.0) | 21 (0.0) |

Abbreviations: N, number; AST, aspartate aminotransferase; ALT, alanine aminotransferase; Total-C, total cholesterol; HDL-C, high-density lipoprotein cholesterol; LDL-C, low-density lipoprotein cholesterol; FBS, fasting blood glucose.

**Supplementary Table 3.** Results of risk of Gout considering the TG/HDL ratio as a time-dependent covariate.

| Variables | Total  N=300,109 | | DM  N=67,371 | | non-DM  N=232,738 | |
| --- | --- | --- | --- | --- | --- | --- |
|  | Model 1 HR (95% CI) | Model 2 HR (95% CI) | Model 1 HR (95% CI) | Model 2 HR (95% CI) | Model 1 HR (95% CI) | Model 2 HR (95% CI) |
| TG/HDL ratio | 1.018 (1.015, 1.02) | 1.014 (1.011, 1.017) | 1.019 (1.011, 1.028) | 1.018 (1.008, 1.028) | 1.017 (1.014, 1.02) | 1.014 (1.011, 1.017) |
| Age, years |  |  |  |  |  |  |
| < 65 | ref | ref | ref | ref | ref | ref |
| ≥ 65 | 0.597 (0.577, 0.617) | 0.480 (0.463, 0.498) | 0.580 (0.542, 0.621) | 0.505 (0.470, 0.543) | 0.589 (0.566, 0.612) | 0.469 (0.450, 0.489) |
| Sex |  |  |  |  |  |  |
| Female | ref | ref | ref | ref | ref | ref |
| Male | 1.371 (1.325, 1.418) | 1.446 (1.382, 1.514) | 1.192 (1.111, 1.279) | 1.288 (1.175, 1.413) | 1.408 (1.355, 1.464) | 1.496 (1.420, 1.576) |
| Body mass index (kg/m2) |  |  |  |  |  |  |
| < 25 |  | ref |  | ref |  | ref |
| ≥ 25 |  | 1.090 (1.054, 1.128) |  | 1.077 (1.007, 1.153) |  | 1.093 (1.051, 1.137) |
| Household income |  |  |  |  |  |  |
| Low |  | ref |  | ref |  | ref |
| High |  | 1.038 (1.002, 1.075) |  | 1.053 (0.981, 1.130) |  | 1.031 (0.991, 1.073) |
| Smoking status |  |  |  |  |  |  |
| Never |  | ref |  | ref |  | ref |
| Former |  | 0.950 (0.905, 0.996) |  | 0.934 (0.850, 1.026) |  | 0.955 (0.904, 1.010) |
| Current |  | 0.886 (0.836, 0.940) |  | 0.830 (0.741, 0.930) |  | 0.912 (0.852, 0.977) |
| Alcohol consumption (days/week) |  |  |  |  |  |  |
| None |  | ref |  | ref |  | ref |
| 1-2 times |  | 0.986 (0.944, 1.030) |  | 0.965 (0.881, 1.057) |  | 0.993 (0.945, 1.045) |
| 3-4 times |  | 1.138 (1.070, 1.210) |  | 1.066 (0.942, 1.206) |  | 1.164 (1.084, 1.251) |
| ≥ 5 times |  | 1.238 (1.146, 1.336) |  | 1.069 (0.916, 1.248) |  | 1.304 (1.193, 1.425) |
| Regular physical activity (days/week) |  |  |  |  |  |  |
| None |  | ref |  | ref |  | ref |
| 1-4 days |  | 0.913 (0.873, 0.955) |  | 0.900 (0.823, 0.984) |  | 0.918 (0.872, 0.967) |
| ≥ 5 days |  | 0.917 (0.876, 0.960) |  | 0.942 (0.861, 1.031) |  | 0.910 (0.863, 0.960) |
| Hyertension |  | 1.182 (1.139, 1.226) |  | 1.167 (1.077, 1.266) |  | 1.181 (1.133, 1.231) |
| Diabetes mellitus |  | 0.807 (0.775, 0.841) |  |  |  |  |
| Dyslipidemia |  | 1.095 (1.055, 1.137) |  | 1.014 (0.933, 1.102) |  | 1.120 (1.074, 1.169) |
| Renal disease |  | 1.759 (1.673, 1.849) |  | 1.797 (1.658, 1.948) |  | 1.734 (1.627, 1.848) |
| Liver disease |  | 1.279 (1.228, 1.332) |  | 1.308 (1.217, 1.405) |  | 1.270 (1.209, 1.334) |
| Charlson comorbidity index |  |  |  |  |  |  |
| 0 |  | ref |  | ref |  | ref |
| 1 or more |  | 2.288 (2.186, 2.396) |  | 2.201 (1.851, 2.618) |  | 2.306 (2.199, 2.419) |

Abbreviations: TG, triglyceride; HDL, high-density lipoprotein; N, number; HR, hazard ratio; CI, confidence interval; DM, diabetes mellitus. The estimated HR (95% CI) was derived from the time-dependent Cox regression model, with the TG/HDL ratio being considered as a time-dependent covariate, and the last time point of follow-up information was used for other adjustment variables.

**Supplementary Table 4.** Risk of Gout based on the average TG/HDL ratio quartile during the follow-up period.

| Variables | Total  N=300,109 | | DM  N=67,371 | | non-DM  N=232,738 | |
| --- | --- | --- | --- | --- | --- | --- |
|  | Model 1 HR (95% CI) | Model 2 HR (95% CI) | Model 1 HR (95% CI) | Model 2 HR (95% CI) | Model 1 HR (95% CI) | Model 2 HR (95% CI) |
| average TG/HDL ratio |  |  |  |  |  |  |
| Q1 | ref | ref | ref | ref | ref | ref |
| Q2 | 1.125 (1.070, 1.183) | 1.068 (1.016, 1.124) | 1.047 (0.948, 1.157) | 1.016 (0.919, 1.123) | 1.106 (1.044, 1.171) | 1.054 (0.994, 1.116) |
| Q3 | 1.225 (1.166, 1.287) | 1.120 (1.065, 1.178) | 1.094 (0.992, 1.207) | 1.038 (0.940, 1.146) | 1.198 (1.132, 1.268) | 1.104 (1.042, 1.169) |
| Q4 | 1.467 (1.399, 1.538) | 1.299 (1.236, 1.366) | 1.334 (1.214, 1.465) | 1.253 (1.138, 1.379) | 1.456 (1.379, 1.538) | 1.305 (1.234, 1.381) |
| Age, years |  |  |  |  |  |  |
| < 65 | ref | ref | ref | ref | ref | ref |
| ≥ 65 | 0.593 (0.574, 0.613) | 0.481 (0.464, 0.499) | 0.586 (0.548, 0.627) | 0.509 (0.473, 0.547) | 0.586 (0.563, 0.609) | 0.469 (0.450, 0.489) |
| Sex |  |  |  |  |  |  |
| Female | ref | ref | ref | ref | ref | ref |
| Male | 1.313 (1.268, 1.359) | 1.417 (1.354, 1.483) | 1.169 (1.089, 1.255) | 1.274 (1.161, 1.397) | 1.347 (1.295, 1.402) | 1.459 (1.385, 1.538) |
| Body mass index (kg/m2) |  |  |  |  |  |  |
| < 25 |  | ref |  | ref |  | ref |
| ≥ 25 |  | 1.064 (1.028, 1.102) |  | 1.066 (0.996, 1.142) |  | 1.063 (1.021, 1.106) |
| Household income |  |  |  |  |  |  |
| Low |  | ref |  | ref |  | ref |
| High |  | 1.039 (1.003, 1.076) |  | 1.054 (0.982, 1.131) |  | 1.031 (0.991, 1.074) |
| Smoking status |  |  |  |  |  |  |
| Never |  | ref |  | ref |  | ref |
| Former |  | 0.942 (0.898, 0.988) |  | 0.930 (0.846, 1.022) |  | 0.947 (0.895, 1.001) |
| Current |  | 0.866 (0.816, 0.918) |  | 0.818 (0.729, 0.916) |  | 0.888 (0.829, 0.952) |
| Alcohol consumption (days/week) |  |  |  |  |  |  |
| None |  | ref |  | ref |  | ref |
| 1-2 times |  | 0.99 (0.947, 1.034) |  | 0.966 (0.883, 1.058) |  | 0.997 (0.948, 1.049) |
| 3-4 times |  | 1.147 (1.078, 1.220) |  | 1.067 (0.943, 1.208) |  | 1.176 (1.095, 1.264) |
| ≥ 5 times |  | 1.249 (1.157, 1.349) |  | 1.075 (0.921, 1.254) |  | 1.318 (1.206, 1.440) |
| Regular physical activity (days/week) |  |  |  |  |  |  |
| None |  | ref |  | ref |  | ref |
| 1-4 days |  | 0.918 (0.878, 0.960) |  | 0.902 (0.825, 0.986) |  | 0.924 (0.877, 0.973) |
| ≥ 5 days |  | 0.926 (0.885, 0.970) |  | 0.949 (0.867, 1.038) |  | 0.920 (0.872, 0.970) |
| Hyertension |  | 1.172 (1.129, 1.216) |  | 1.163 (1.073, 1.261) |  | 1.170 (1.122, 1.220) |
| Diabetes mellitus |  | 0.789 (0.757, 0.822) |  |  |  |  |
| Dyslipidemia |  | 1.082 (1.042, 1.123) |  | 1.007 (0.926, 1.095) |  | 1.105 (1.059, 1.152) |
| Renal disease |  | 1.747 (1.662, 1.837) |  | 1.786 (1.648, 1.937) |  | 1.722 (1.616, 1.836) |
| Liver disease |  | 1.280 (1.229, 1.334) |  | 1.308 (1.217, 1.405) |  | 1.271 (1.210, 1.336) |
| Charlson comorbidity index |  |  |  |  |  |  |
| 0 |  | ref |  | ref |  | ref |
| 1 or more |  | 2.292 (2.190, 2.399) |  | 2.197 (1.848, 2.613) |  | 2.311 (2.204, 2.424) |

Abbreviations: TG, triglyceride; HDL, high-density lipoprotein; N, number; HR, hazard ratio; CI, confidence interval; Q, quartile; DM, diabetes mellitus. The estimated HR (95% CI) was derived from the conventional Cox regression model, wherein the TG/HDL ratio was considered as the quartiles of its average during the follow-up period, and the last time point of follow-up information was used for other adjustment variables.

**Reference**

1. Park, H., Chang, Y., Lee, H., Hong, I., & Song, T. J. (2023). Association of total cholesterol variability with risk of venous thromboembolism: A nationwide cohort study. Plos one, 18(8), e0289743.

2. Chang, Y., Kang, M. K., Park, M. S., Leem, G. H., & Song, T. J. (2023). Resolved Proteinuria May Attenuate the Risk of Heart Failure: A Nationwide Population-Based Cohort Study. Journal of Personalized Medicine, 13(12), 1662.

3. Park, H., Chang, Y., Lee, H., Hong, I., & Song, T. J. (2023). Association of total cholesterol variability with risk of venous thromboembolism: A nationwide cohort study. Plos one, 18(8), e0289743.

4. Lee, S., Chung, W., & Hyun, K. R. (2011). Socioeconomic costs of liver disease in Korea. The Korean Journal of Hepatology, 17(4), 274.

5. Sundararajan, V., Henderson, T., Perry, C., Muggivan, A., Quan, H., & Ghali, W. A. (2004). New ICD-10 version of the Charlson comorbidity index predicted in-hospital mortality. Journal of clinical epidemiology, 57(12), 1288-1294.
